# Feeling Free and Having an Authentic Inner Compass as Important Aspects of the Need for Autonomy in Emerging Adults’ Interactions With Their Mothers

**DOI:** 10.3389/fpsyg.2021.635118

**Published:** 2021-10-05

**Authors:** Avi Assor, Rinat Cohen, Ohad Ezra, Shi Yu

**Affiliations:** ^1^Educational Psychology Program, Department of Education, Ben Gurion University of the Negev, Beersheba, Israel; ^2^Department of Psychology, Chinese University of Hong Kong, Shenzhen, China

**Keywords:** need for autonomy, authentic inner compass, emerging adults, relationship with parents, freedom

## Abstract

Based on past theorizing and research, we posited that there are two kinds of specific experiences that contribute to the satisfaction of the general need for autonomy in emerging adults, as reflected in volitional, self-endorsed, actions. These experiences are: (1) feeling free, and (2) having a valid authentic inner compass (AIC). In the first study testing this hypothesis, college students in Israel (*n* = 163, mean age = 21.33), and in China (*n* = 72, mean age = 23.67) completed measures assessing experiences of freedom and having a valid AIC during contacts with mothers, extent of volitional contacts with mothers, and vitality during contacts with mothers. Confirmatory factor analyses and invariance analysis supported the validity of the measures, and their equivalence across cultures. In the Israeli sample, students also completed a measure assessing the extent to which the sense of having a valid AIC during contacts with one’s mother is based on intrinsic aspirations and goals. As expected, across cultures, participants distinguished between experiences of (a) having a valid AIC, and (b) feeling free. The findings also suggest that experiences of freedom and of having a valid AIC during contacts with mothers are associated with extent of volitional contacts with mother, and subsequent vitality during these contacts. Also as expected, experiencing a valid AIC during contacts with mother was associated with volitional contacts with her and subsequent vitality - only when the AIC was based on intrinsic aspirations. The results suggest that in assessing people’s sense of AIC, it is important to consider the content of the aspirations and goals on which this sense is based. The findings are consistent with the view that feeling free, and having a valid AIC are two specific autonomy experiences which promote a more global experience of need autonomy satisfaction, as indicated by feelings of volitional and self-endorsed action.

## Introduction

Self-determination theory (SDT; Deci and Ryan, 2000; Ryan and Deci, 2017) posits that humans have a basic psychological need for autonomy, whose satisfaction is critical to their socio-emotional functioning and well-being. In a seminal paper on SDT’s conception of needs (Deci and Ryan, 2000) describe the need for autonomy as an organismic desire *“toward **self-regulation** of action and coherence” (p. 253), and as* a need “*to **self-organize and regulate** one’s own behavior (and avoid **heteronomous** control; p. 252)*. In a chapter on psychological needs in Ryan and Deci’s (2017) book on self-determination theory, the authors note the importance of the need of autonomy as “*a vehicle through which the **organization** of the personality proceeds, and through which other psychological needs are actualized*,” in ways that feel volitional and fully self-endorsed (p. 97).

Following the above emphasis on self-direction and organization-enabling actions that feel volitional and self-endorsed, suggested that in order to feel that their need for autonomy is optimally satisfied, people need to have three kinds of specific volitional experiences: (1) freedom (2) experiences of having a valid authentic inner compass (AIC), and (3) behavioral congruence and self-realization.

Freedom refers to the perception that you are not coerced or pressured to do something, and in addition you are free to explore what may be interesting, satisfying, or meaningful for you. The experiences of coercion and freedom can be based on inputs coming from other people or contexts, but can also originate from internal sources. An internal source of pressure can be an extreme desire to win or maintain the love and appreciation of parents (Assor et al., 2004). An internal source of freedom can be a non-defensive openness to experience, or absence of ego-involvement.

Sense of having an AIC refers to the experience of having aspirations, values, goals, and preferences that are intrinsically rather extrinsically oriented, feel deeply authentic, and function like an “authentic inner compass” (AIC). That is, intrinsically-oriented self-guides that inform us on *what is truly important for us, and are experienced as worthy and valuable, personally meaningful, reasonable, and enabling self-fulfillment*. As these self-guides are posited to function as a more deeply authentic inner compass if they are intrinsically oriented, it is important to clarify the terms “intrinsic” and “extrinsic” in this context. SDT (e.g., Ryan et al., 1996; Ryan and Deci, 2017) distinguishes between intrinsic and extrinsic life aspirations. The theory further posits that intrinsic life aspirations can be experienced as deeply authentic, because they direct us to act in ways that promote deep satisfaction of our basic psychological needs. In contrast, extrinsically oriented life aspirations cannot be experienced as fully authentic, because they only provide very limited and partial (compensatory, substitute) need satisfaction.

SDT-based research has focued so far mainly on three intrinsic life aspirations (meaningful relationship, community contribution, and personal growth), and three extrinsic aspirations (power, prestige, and great wealth). However, following Assor (2018), we posit that intrinsic life aspirations and specific goals can also have other contents, as long as they reflect authentic organismic inclinations that do not undermine basic need satisfaction. For example, developing specific individual interests, finding a work or a life style that is consistent with one’s temperament inclinations (e.g., Assor et al., 2020a), or sexual orientation.

Empirical research has provided considerable support to SDT’s proposition that aspirations, values and goals are experienced as more autonomous, and are more satisfying and growth-promoting when they are intrinsic rather than extrinsic (Sheldon et al., 2004; Ryan and Deci, 2017). Based on these research findings and theoretical assumptions, Assor (2018) posited that reports of having values, aspirations and goals that are personally important and meaningful, worthy and reasonable, are more likely to reflect a deeply authentic inner compass experience to the extent that these self-guides are intrinsically-oriented.

Because the construct of AIC includes reference to the concept of intrinsic aspirations, it is important to distinguish it from this concept. Sense of AIC refers to the ***global and direct experience*** that one has value aspirations, and goals that mostly intrinsically oriented, and are truly important and valuable, personally meaningful, and likely to allow self-fulfillment. This global and direct experience is assumed to contribute to the feeling of being autonomous (i.e., feeling capable of true self direction because we know what is truly important to us). While this global AIC experience may be correlated with endorsing the three specific intrinsic life aspirations posited by SDT (Kasser and Ryan, 1996), it cannot be reduced to the endorsement these three aspirations, for two reasons.

First, the self-guides constituting one’s AIC also include specific concrete goals that are different from general long-term life aspirations. For example, being able to realize a specific interest or life style (e.g., engagement in sports activities), finding work that fits one’s temperament inclinations (e.g., active work in nature, in small familiar teams, away from large crowds). Importantly, starting from adolescence, a firm AIC is assumed to include not only general life aspirations, but also specific goals (Assor, 2018).

Another source of difference between a global sense of AIC and intrinsic life-aspirations as conceptualized and examined in SDT, is that the specific intrinsic aspirations underlying the experience of having an AIC *do not necessarily overlap with the specific intrinsic aspirations examined in most SDT research*. Thus, *the AIC experience of a given individual can reflect only some of the life-aspirations identified in SDT*, and may also include additional aspirations, and specific goals, not included in current SDT research on intrinsic aspirations. For example, being able to live openly according to one’s sexual orientation, or developing a career in which one feels a sense of competence and interest. While some of these self-guiding aspirations or goals may be correlated with general life-aspirations identified by SDT, they are far from being the same.

According to Assor (2018, and see also Assor et al., 2020b), beginning in adolescence, the sense of having enduring authentic self-guides is posited to be an important aspect of the experience of autonomy need satisfaction because it enables us to feel that we can **self-organize** and **direct** our behavior toward destinations that feel **self-endorsed and volitional**. And, as noted by Assor (2018), and in the SDT quotes above, the striving to self-organize and self-direct in ways that allow self-actualization and sense of volition is a core feature of the need for autonomy. When we do not have such action- and decision- guiding inner compass, we are likely to feel confused and not capable of self-endorsed and volitional, self-direction, because we do not know what actions to choose. Consequently, we may prefer to escape from freedom even when we are relatively free to direct our lives (Fromm, 1941).

Sense of having an AIC as an important aspect of the need for autonomy is likely to become particularly significant in adolescence and emerging adulthood, when youth have to decide about future plans, as well as react to situations involving social pressures and serious moral or personal implications (e.g., Assor et al., 2020b,c). Therefore, emerging adults may have a particularly strong need to be in relationships that sustain and validate their confidence in the merit of the intrinsic aspirations and goals constituting their inner compass.

Similarly, and perhaps even more so, they have a need to avoid relationships providing “toxic” inputs that undermine their sense of AIC, and as a result, their capacity to strive, imagine, plan and act in ways that feel autonomous (i.e., volitional and self-endorsed).

Research on the AIC construct has already shown that the experience of having an AIC has a positive effect on expected outcomes of need satisfaction, such as well-being and optimal functioning. Thus, having an AIC was found to contribute to central indicators of well-being such as vitality, low levels of depression, sense of meaning, and self-esteem (Soenens et al., 2016; Yu et al., 2018a; Assor et al., 2020b, 2021; Russo-Netzer and Shoshani, 2020). The experience of having an AIC was also found to predict the capacity to stand by one’s self-endorsed values in the face of social pressure (Soenens et al., 2016; Assor et al., 2020c). Notably, these effects were observed in youth belonging to very different cultures (Chinese, Jews, Bedouins, and Belgians).

While these results support the merit of the AIC construct, there are two aspects of the theorizing concerning the experience of AIC that were not examined so far, and are the focus of the present study. First, no study to date has examined the proposition that: (a) the experience of having an AIC is distinct from the experience of freedom, and (b) both experiences are uniquely associated with general autonomy need satisfaction, as indicated by volitional and self-endorsed action, and with vitality (a major expected outcome of need autonomy satisfaction).

Second, no study to date has focused on the content of the aspirations, values, and goals on which people’s sense of inner compass is based. Thus, past research has assessed the sense of AIC *via* reports concerning the extent to which people have values, aspirations and goals felt to be truly and personally important, worthy, and self-fulfilling. Research has shown that this measure of sense of AIC is associated with aspirations and goals that are more intrinsic than extrinsic (Assor et al., 2015; Vermote et al., 2018). Yet, as the association is not very strong, we cannot assume that the past measure of AIC refers only to intrinsically oriented self-guides. Therefore, it appears that we can obtain a more accurate assessment of the experience of AIC, if we take into consideration both the extent to which people have values goals and aspirations described as truly important and self-fulfilling, and the extent to which these self-guides are intrinsically rather than extrinsically oriented. Accordingly, we assume that the sense of inner compass that emerging adults would like to develop and validate can be considered deeply authentic only if it is based on aspirations and goals that are more intrinsic than extrinsic.

Theoretically, an inner compass that is more deeply authentic is assumed to contribute more to volitional action and vitality. Hence, we expected that reports of experiencing a content-free AIC (feeling that one has an AIC without specifying its content), will be more strongly associated with volitional action and vitality, when the inner compass is intrinsically oriented (i.e., based on aspirations and goals that are more intrinsic than extrinsic).

### Experiences of Freedom and Having a Valid AIC When Spending Time With Mother as Predictors of Volitional Contacts With Mother and Subsequent Vitality During These Contacts

Considerable research has shown that people can experience different levels of need satisfaction in different relationships (La Guardia et al., 2000). Therefore, to obtain accurate and sensitive indicators of need satisfaction experiences, it is reasonable to assess these experiences in the context of specific relationships. In the present study we chose the context of spending time with one’s mother (*via* actual or virtual contact). The participants were college students, who do not live with their mothers.

Research has shown that maintaining close and supportive relationship with mothers is important for young adults and contributes to their adjustment (Ryan and Lynch, 1989; Aquilino, 2006; Kenny and Sirin, 2006; Buhl, 2007; Fingerman et al., 2012; Guarnieri et al., 2015). Research has also shown that many emerging adults can benefit from close relationships with their mothers also when they do not live with them (Kins et al., 2009, 2014; Mattanah et al., 2011).

However, there is substantial evidence that relationships with parents are optimally beneficial and desirable for adolescents and emerging adults only to the extent that children feel that their need for autonomy is satisfied (rather than frustrated) in their contacts and interactions with their parents (Ahmad et al., 2013; Fousiani et al., 2016; Inguglia et al., 2018).

As autonomy need satisfaction in contacts with mothers is a major determinant of the extent to which emerging adults sustain this needed, growth-promoting, relationship, it is important to understand the specific autonomy need satisfaction experiences that contribute to emerging adults’ motivation to maintain contacts with mothers. Yet, the research on the importance of autonomy need satisfaction in emerging adults’ relationships with their parents did not address several important issues, which the present study begins to examine. First, extant studies did not examine the extent to which autonomy need satisfaction in contacts and interactions with parents promote offspring’s inclination to maintain volitional, autonomously motivated, contacts and interactions with their parents, and the extent to which such contacts are vitalizing for children. Ryan et al. (2005) found that college students who experience basic need satisfaction in their relationship with their parents are more inclined to rely on their parents emotionally. Lynch (2013) found that the trait of autonomy and perceived parent autonomy support were associated with the tendency to rely emotionally on others. However, these studies did not examine specifically volitional motivation to spend time with one’s parents, nor did they directly assess the experience of autonomy need satisfaction during contacts with parents.

Second, this research did not examine the distinct associations of the two aspects of autonomy need satisfaction posited by Assor (2018) - having a valid AIC, and freedom - with positive relational and affective outcomes. Recent research (Assor et al., 2021) has shown that a widely used measure of autonomy need satisfaction (Chen et al., 2015) does not capture the experience of having AIC. Furthermore, both having an AIC and the Chen et al. (2015) measure had unique positive effects on important well-being indicators (vitality and low depression).

As already noted, Assor (2018) posits that both freedom and having AIC are important need-satisfying experiences. Therefore, we expected that feeling that during contacts with parents one’s sense of AIC is strengthened and validated (rather than weakened and invalidated), and feeling that during these contacts one is free (and not controlled), will be both associated with clear indicators and outcomes of autonomy need satisfaction; namely, feelings of volition during contacts with parents, which will then promote vitality during interactions with parents.

The proposition that emerging adults need to feel a sense of freedom (and lack of coercion) in their relationships with parents is shared by important theoretical and empirical writings (e.g., Erikson, 1968; Marcia, 1988), especially in relation to the task of forming important identity commitments. One of the major life tasks that late adolescents and emerging adults face is developing future commitments (Erikson, 1968; Marcia, 1988; Luyckx et al., 2011). In order to develop satisfying, growth-promoting commitments, adolescents need to feel that their parents respect their need to freely explore ideas, values, and aspirations, goals, social contexts, relationships, career options, and life styles. And, indeed, the capacity to engage in free and open exploration was found to promote commitments that are associated with well-being and adjustment (Meeus, 2011; Schwartz et al., 2011).

Hence, we hypothesized that an emerging adults who feel that in their interactions with parents they are free to explore different ideas and directions (rather than being pressured to think and act in parentally expected ways) will be autonomously motivated to spend time with their parents. Furthermore, because their striving to feel free and not pressured is satisfied in these contacts, these emerging adults will also feel vitalized when spending time with parents.

Yet, emerging adults need more than freedom; they also need to develop and maintain aspirations, values and goals that allow them to form commitments and engagements that feel authentic (AIC), satisfying, and worth pursuing (Assor et al., 2020b). As emerging adolescents are often not fully sure about the aspirations and goals constituting the inner compass they have developed, they need to feel that their AIC remains intact, and preferably feels even more valid, when they are in contact with significant others, including parents. Put differently, emerging adults need to feel that during and after interactions with their parents, they maintain or increase their confidence in the merit and authenticity of the aspirations and goals constituting their AIC. At the minimum, they need to feel that interactions with parents do not undermine their sense of having worthy and reasonable, direction-giving and action-guiding, AIC.

### Examining the Distinction Between Freedom and AIC Experiences in Widely Different Cultures

In SDT, an important criterion for considering a psychological construct as a basic need is that this construct is associated, across *widely different cultures*, with well-being and optimal functioning qualities expected to emerge as a result of basic need satisfaction. Therefore, in the present study, we examined if the strivings to feel free and to have an AIC are experienced as distinct and as having optimal relational and well-being correlates in two widely different cultures: Chinese and Jewish Israeli. Specifically, we examined whether emerging adults in both cultures distinguish between feeling that they have a firm and valid AIC and feeling free during their interactions with their parents. In addition, we also examined whether these two distinct experiences during contacts with parents are associated with feelings of volition during these contacts, and subsequent vitality in this context.

The two cultures chosen for the present study are suitable to examine hypotheses about the universality of basic needs and their components, because they differ widely in the importance they assign to freedom and authenticity. While the Jewish culture (except for an ultra-orthodox religious minority) tends to espouse the value of self-direction and autonomy (e.g., Oyserman, 1993; Cohen, 2006, 2007; Schwartz, 2009; Weinstock, 2011), this is not the case for the Chinese culture (e.g., Hsu, 1981; Chao, 1994; Triandis, 2018; Slote and DeVos, 1998; Schwartz, 2009). Moreover, Chinese education, resting on Confucian and socialist philosophies (Levenson, 1972; Tweed and Lehman, 2002), is likely to view the development of a personal, inward-oriented AIC as undesirable because it might lead to breach of dominant social norms. Yet, because we consider the experience of feeling free and having an AIC as basic universal components of the need for autonomy, we posited that the two experiences will have positive relational and well-being correlates also in China.

Consistent with this view, previous studies with Chinese and Jewish Israeli youth and emerging adults did show that, in both cultures, having an AIC was associated with well-being indicators such as vitality and self-congruence (Assor et al., 2015, 2020b, 2021). Previous studies have also mostly supported the important role of autonomy support from parents in Israel and in China (e.g., Roth et al., 2009; Lekes et al., 2010). However, no study to date has examined the specific experiences of freedom and of having AIC in emerging adults’ relationships with parents and their relational and well-being correlates. Given the absence of data on this issue, the present study was undertaken. As freedom and having AIC are assumed to constitute basic universal components of the need for autonomy, we hypothesized that both - feeling free and feeling that one has a valid AIC during contact with parents - are associated with volitional (autonomous) contacts with parents, and subsequent vitality during such contacts.

### The Present Study

In an attempt to explore the cross-cultural importance of experiences of freedom and of having a valid AIC in relationships with parents, we collected data from two samples of emerging adults in two countries. The first sample was comprised of Chinese emerging adults, and examined a relatively simple model in which experiences of feeling free and experiencing one’s AIC as valid during contacts with mother predicts volitional contacts with her, and subsequent vitality during these contacts. As the sense of volition is a primary marker of autonomy need satisfaction, we used an expanded measure of volitional action including direct indicators of volition and lack of volition, in addition to customary indicators of volitional action drawn from the widely used measure of autonomously motivated action (e.g., Ryan and Connell, 1989).

Then, a second sample in Israel was used to replicate the basic model tested in China, and then test a more complex model, in which the effect of having an AIC on volitional contact with mother (and subsequent vitality), is moderated by the extent to which participants’ AIC is intrinsically oriented. The more complex model is based on the assumption that sense of having an inner compass is more deeply authentic, and hence contributes more to volitional action, when it is based on intrinsic values, aspirations and goals.

### Hypotheses

1.Emerging adults will distinguish between experiences of (a) a general [content free] sense of having a valid AIC, and (b) feeling free during interactions and contacts with their mothers.
2.Both experiences will be associated with volitional contacts with mother, and subsequent vitality during these contacts.
3.Reports on experiencing a content-free AIC (feeling that one has a valid AIC without specifying its content) during contacts with mother, will be more strongly associated with volitional contacts with mother, and subsequent vitality, when the experience of having an AIC is based on aspirations and goals that are more intrinsic than extrinsic.

## Materials and Methods

### Participants and Procedure

Participants were Israeli and Chinese emerging adults. The Chinese sample consisted of 72 college students in Beijing. Participants’ age ranged from 19 to 30 (mean age = 21.33, *SD* = 1.84), and 70% of the sample were females. The Israeli sample consisted of 163 college students. Participants’ age ranged from 20 to 31 (mean age = 23.67, *SD* = 1.49), and 72% of the sample were females. The institutional review board (IRB), and participants approved the survey administration in both of the samples. Students chose to participate in the study as part of psychology or education courses. Questionnaires were administered by an unknown research assistant, who assured participants that their responses would be confidential and anonymous.

### Measures

All the Likert type measures in this study were assessed by seven-point scale ranging from not true at all (1) to very true (7). It is important to note that, although all the scales were adapted from extant measures, we assessed their psychometric adequacy and cross-cultural equivalence *via* a multi-group structural equations measurement model.

#### Experiencing One’s AIC as Valid During Contacts With Mother (A General Content-Free Measure)

This variable was assessed in both the Chinese and the Israeli sample. In this measure, participants were asked to write a description of an aspiration or a goal that is presently truly important to them, and with which they truly identify. Following this open response, participants were asked to respond to three items indicating experiencing one’s AIC as valid during contacts with mother, and three items indicating experiencing ones AIC as not valid during contacts with mother.

Items assessing the experience of having a valid AIC asked participants to indicate whether when being with mom or talking with her on the aspiration or goal they described – they felt more confident that their aspiration or goal: (1) is valuable or important (2) more satisfying, self-fulfilling, or personally meaningful, and (3) more reasonable. Items assessing the experience of AIC as not valid asked participants to indicate whether when being with mom or talking with her on the aspiration or goal they described – they felt less confident that their aspiration or goal: (1) is valuable or important (2) is satisfying, self-fulfilling, meaningful, and (3) is reasonable. The items assessing AIC as not valid were reversed. Cronbach’s alpha for this scale was 0.74 in the Israeli sample and 0.80 in the Chinese sample.

The measure was first written in Hebrew, then, translated to English and then to Chinese, and then back-translated to English and Hebrew. More recent studies used these items to construct an AIC scale that is not based on an initial open response, and refers to the general experience of having an AIC. This later scale produces a pattern of correlates and outcomes supporting the construct validity of the items (e.g., Soenens et al., 2016; Yu et al., 2018a,b; Assor et al., 2020b,c, 2021; Russo-Netzer and Shoshani, 2020). Following an idiographic approach, the scale based on an initial open response was preferred in this study because it may allow us to access the aspirations and goals that are especially relevant to participants. In addition, it allows us to classify the extent to which their sense of having an AIC is based on intrinsic aspirations. It is important to note that, unlike the next measure to be described, the general AIC scale is a content-free measure of sense of having AIC.

#### Intrinsically-Oriented AIC Content

This measure assessed the extent to which the sense of AIC validated by mothers was intrinsically rather than extrinsically oriented. As already noted, we assumed that an AIC that is based mainly on intrinsic aspirations, is more authentic, and therefore hypothesized that it will have a particularly strong association with volitional and vitalizing contact with mother. The extent to which participants’ AIC was based on aspirations and goals that are more intrinsic than extrinsic was determined by a coding system based on SDT’s conceptualization of intrinsic vs. extrinsic life goals (e.g., Ryan et al., 1996; Ryan and Deci, 2017), but including also aspirations that although likely to support basic psychological needs are not explicitly included in the goal categories appearing in Ryan et al. (1996). In addition to the open description of the aspiration or the goal, we also relied on a question that followed the aspiration or goal description, and asked participants to write the reasons why the goal was important to them.

For example, aspirations were coded as highly intrinsic if they clearly referred to having close and intimate relationships, finding studies or work that is interesting or allows development of knowledge and skills, or community contribution, and the reasons also referred to intrinsic rather than extrinsic aspirations. For example, developing knowledge and skills because it is satisfying and can help the community, as opposed to developing knowledge and skills because it can help your gain much wealth and status. Aspirations were coded as extrinsic if they referred to gaining a great deal of power and wealth, a great deal of power or wealth, prestige, and doing much better than others in various domains. 59% of the aspirations were coded as highly intrinsic, and there were very few participants who described clearly extrinsic aspirations. Therefore, we distinguished only between aspirations that are highly intrinsic and aspirations that are not highly intrinsic. This measure was examined only in the Israeli sample, because of its exploratory nature. Inter rater reliability was assessed on 30 participants, and was satisfactory (Cohen’s Kappa = 0.81).

#### Experiencing Freedom During Contacts With Mother

The scale assessing this variable was designed to capture the construct of experiencing freedom as conceptualized by Assor (2018) and in this study. It includes two items assessing experiences of freedom and two items assessing experiences of coercion (internal and external). The items were adapted from extant need autonomy scales (La Guardia et al., 2000; Weinstein and Ryan, 2010). A sample coercion item is: “When I am with my mom or when talking to her - I feel controlled and pressured to be certain ways.” A sample freedom item is: “When I am with my mom or when talking to her - I feel that I can do what I really want to do.” Cronbach’s alpha for this scale was 0.81 in the Israeli sample and 0.74 in the Chinese sample.

#### Volitional Contacts With Mother

The extent to which participants felt that their contacts and interactions with mothers were volitional (autonomously motivated) was assessed by items similar to those of close relationship motivation scales (Blais et al., 1990; Gaine and LaGuardia, 2009), and similar self-regulation motivation scales (e.g., Ryan and Connell, 1989; Assor et al., 2009). Given the importance of the experience of true volition as an indicator of general need autonomy satisfaction, we also added several items assessing sense of volition vs. absence of such volition.

Consistent with Blais et al. (1990) and Gaine and LaGuardia (2009) relational motivation scales, our global index of volitional contacts with mother, was created by subtracting an indicator representing nonvoluntary contacts (controlled, a-motivated, and resistant contacts) from an indicator representing volitional contacts. The measure indicating **nonvoluntary contacts** was comprised of five items capturing contacts driven by **external and introjected motives** (e.g., “I spend time with my mom or talk to her, because I do not want other people to think that I am a bad son”), and five items directly assessing **lack of** **volition** to maintain contact; that is, a-motivation and resistance to maintain contacts with mothers (“If I had a choice - I would visit my mom only rarely”; “I often feel that I do not really want to spend time with my mom”). Cronbach’s alpha was 0.89 in the Israeli sample, and 0.92 in the Chinese sample.

The measure indicating presence of **volitional contacts** included seven items capturing contacts due to **intrinsic and identified motives** (e.g., “I spend time with my mom or talk to her because I enjoy being with her”), and three items assessing **volition** directly (e.g., “I spend time with my mother because I **really want to** and NOT because **I have to**”). Cronbach’s alpha was 0.94 in both samples. The correlation between the two scales was negative and significant (Israeli: *r* = −0.80, *p* < 0.001; Chinese: *r* = −0.51, *p* < 0.001). As noted above, we created an index of overall volitional contacts with mother by subtracting the scale assessing non-voluntary contact from the scale assessing volitional contact.

#### Vitality During Contacts With Mother

Participants’ experience of vitality when spending time with mother was measured using a 5-item scale based on Ryan and Frederick’s (1997) vitality scale. Sample items include “When I am with my mother, I feel alive and vital,” and “When I am with my mother, I feel exhausted.” Cronbach’s alpha for this scale was 0.89 for the Israeli sample, and 0.92 for the Chinese sample.

### Measurement Model

We tested measurement invariance across cultures using Amos24 (Arbuckle, 2006).

Degree of metric and scalar invariance was assessed by comparing, for both analyses, the Chi-square, CFI, and RMSEA values characterizing a model constrained to equivalence vs. an unconstrained model. Non-significant chi-square difference, and a CFI and RMSEA differences not exceeding a value of 0.01 suggest that the models can be viewed as equivalent (Cheung and Rensvold, 2002; Chen, 2007).

First, we calculated a baseline model (the configured model) including four latent factors corresponding to the four variables examined. As experiences of having a valid AIC and of freedom are fairly new constructs and measures, the latent construct representing each variable was assessed by all the items assumed to capture it, without using any parcels. The less innovative constructs of volitional (autonomous) contacts, involuntary (non-autonomous) contacts, and vitality, were assessed by parcels of items which usually capture these constructs. This was done in order to create a reasonable ratio of observed indicators relative to the sample size (Bagozzi and Edwards, 1998; Bandalos and Finney, 2001). Volitional contacts and involuntary contacts were each assessed by two 3-item parcels, and one 4-items parcel. Vitality was assessed by one 3-item parcel, and one 2-item parcel. Each parcel was created by averaging randomly chosen items. The baseline model fit showed an acceptable baseline model with *χ*^2^(246) = 548.79, *p* < 0.001, CFI = 0.91, RMSEA = 0.07. All the factor loadings were significant (*p* < 0.001) and greater than 0.59 in the Israeli sample and 0.56 in the Chinese sample (For all the items loadings see Table 1).

**Table 1:** Results of the measurement model for the Israeli and Chinese samples.

Variable’s name	Number of items	Factor loadings	
		The Israeli sample	The Chinese sample
Experiencing a valid content – free	1	0.79	0.82
AIC during contacts with mother	2	0.62	0.58
	3	0.87	0.94
	4	0.85	0.92
	5	0.64	0.58
	6	0.61	0.57
Experiencing freedom during contacts with mother	1	0.74	0.77
	2	0.83	0.79
	3	0.72	0.56
	4	0.75	0.72
Voluntary contacts	Parcel 1	0.91	0.92
	Parcel 2	0.93	0.79
	Parcel 3	0.93	0.94
Nonvoluntary contacts	Parcel 1	0.62	0.76
	Parcel 2	0.67	0.68
	Parcel 3	0.93	0.94
Vitality during contacts	Parcel 1	0.87	0.70
With mother	Parcel 2	0.81	0.95

Then, multi group analyses were performed. First, we assessed metric invariance across samples by comparing a model in which factor loadings were constrained to be equal and a model in which these loading remained unconstrained. Results revealed that the constrained model fit the data well, the difference between the chi-square values of the two models was not significant (∆*χ*^2^(11) = 10.81, n.s.), and the CFI and RMSEA differences did not exceed the value of 0.01 (∆CFI = 0.01; ∆RMSEA = 0.001). These findings suggest that factor loadings of both groups were invariant.

Next, we assessed scalar invariance. This was done by comparing a model in which both factor loading and intercepts were constrained to be equal across the samples with a model in which only the factor loadings were constrained. Results revealed that the constrained model fit the data well, and the difference between the chi-square values of the two models was not significant (∆*χ*^2^(18) = 17.82, n.s.). In addition, the RMSEA difference did not exceed the recommended value of 0.01 (∆RMSEA = 0.007). However, the CFI difference was a little higher than the recommended value of 0.01 (∆CFI = 0.03), suggesting that there is no clear evidence for scalar invariance. However, given that the indexes of ∆RMSEA and ∆χ^2^ were clearly satisfactory, and ∆CFA did not deviate much from the recommended values, it appears that there is some support for scalar invariance. Yet, given the ∆CFI we obtained, it is important to note that results pertaining to mean differences assuming scalar invariance, should be interpreted with great caution.

To ascertain that items assumed to capture experiences of freedom and experiences of having a valid AIC indeed reflect two different constructs rather than one factor, we compared a measurement model in which freedom and AIC items represented one latent factor, with a model in which these items represented two different structures. The models also included the three additional latent factors examined in the invariance analysis already reported. Results showed that the model in which AIC and freedom items represented two different latent factors fitted the data significantly better than the model in which the AIC and freedom items represented one latent factor, Δ*χ*^2^(6) = 454.04, *p* < 0.001. These findings suggest that experience of having a valid AIC and the experience of freedom are indeed separate constructs. The fit indices of the model in which the AIC and freedom items were represented by one factor where unsatisfactory; *χ*^2^(252) = 948.75, *p* < 0.001, *CFI* = 0.78 and *RMSEA* = 0.13.

## Results

### Data Analysis Plan

We first calculated the descriptive statistics, and the zero-order correlations among research variables. Then, using structural equation modeling (AMOS24; Arbuckle, 2006), we assessed whether volitional contact with mother mediates the relations of experiencing a valid content-free AIC and experiencing freedom during contacts with mother with feeling vitality during contacts with mother. Multi-group analyses were conducted to assess the equivalence of the mediation model across the Israeli and Chinese samples. Finally, in the Israeli sample we used structural equation modeling (AMOS24; Arbuckle, 2006), to assess whether intrinsically oriented AIC (sense of AIC based on intrinsic aspirations) moderated the positive effects of experiencing a valid content-free AIC in contacts with mother on overall volitional contacts with mothers, and subsequent vitality during these contacts.

### Descriptive Statistics and Preliminary Analyses

Descriptive statistics and bivariate correlations are presented in Table 2. As can been seen from the table, for both of samples, experiencing a valid content-free AIC during contacts with mother was positively correlated with experiencing freedom during these contacts. As expected, experiences of both content-free AIC and freedom were positively associated with voluntary contacts and vitality, and negatively associated with nonvoluntary contacts. Fisher Z tests indicated that experiencing a valid content-free AIC had a significantly more negative correlation with nonvoluntary contacts than did experiencing freedom (Fisher Z = 7.10, *p* < 0.001 for the Israeli sample; Fisher Z = 4.63, *p* < 0.001 for the Chinese sample). Other variables did not show consistent differences in correlation magnitude. Table 3 presents T tests comparing differences in mean levels between Chinese and Israelis. As shown in the Table 3, Israeli participants reported higher levels of experiencing content-free AIC, freedom, overall volitional contacts with mother, and voluntary contacts with mother, and lower levels of non-voluntary contacts, than did the Chinese participants. The means were almost identical for vitality during contacts with mother.

**Table 2:** Descriptive statistics and correlation among research variables.

	Mean (*SD*)	1	2	3	4	5	6	7		Israeli sample
1. Experiencing a valid content-free AIC during contacts with mother	5.62 (1.00)	1						
2. Experiencing freedom during contacts with mother	5.46 (1.11)	0.45^**^	1					
3. Overall volitional contacts with mother	3.88 (1.90)	0.51^**^	0.61^**^	1				
4. Voluntary contacts with mother	5.81 (1.06)	0.49^**^	0.59^**^	0.95^**^	1			
5. Nonvoluntary contacts with mother	1.92 (0.95)	−0.47^**^	−0.57^**^	−0.94^**^	−0.80^**^	1		
6. Vitality during contacts with mother	5.18 (1.12)	0.39^**^	0.51^**^	0.72^**^	0.72^**^	−0.65^**^	1	
7. Intrinsically oriented AIC content	1.59 (0.50)	0.04	0.06	0.03	0.003	0.05	0.05	1
	Chinese sample							
1.Experiencing a valid content-free AIC during contacts with mother	5.11 (1.08)	1						
2. Experiencing freedom during contacts with mother	4.98 (1.21)	0.55^**^	1					
3. Overall volitional contacts with mother	2.48 (2.15)	0.46^**^	0.71^**^	1				
4. Voluntary contacts with mother	4.94 (1.32)	0.39^**^	0.49^**^	0.89^**^	1			
5. Nonvoluntary contacts with mother	2.45 (1.15)	−0.40^**^	−0.77^**^	−0.85^**^	−0.51^**^	1		
6. Vitality during contacts with mother	5.19 (1.16)	0.33^**^	0.68^**^	0.75^**^	0.61^**^	−0.71^**^	1	

^*^p < 0.05; ^**^p < 0.01; ^***^p < 0.001.

**Table 3:** Comparisons of mean levels in the Chinese and Israeli samples.

	Israeli sample	Chinese sample	*t*	*P*
	Mean (*SD*)	Mean (*SD*)		
1. Experiencing a valid content-free AIC during contacts with mother	5.62 (1.00)	5.11 (1.08)	2.98	0.003
2. Experiencing freedom during contacts with mother	5.46 (1.11)	4.98 (1.21)	2.95	0.003
3. Overall volitional contacts with mother	3.88 (1.90)	2.48 (2.15)	4.95	<0.001
4. Voluntary contacts with mother	5.81 (1.06)	4.94 (1.32)	5.34	<0.001
5. Nonvoluntary contacts with mother	1.92 (0.95)	2.45 (1.15)	3.64	<0.001
6. Vitality during contacts with mother	5.18 (1.12)	5.19 (1.16)	0.057	0.957

### Tests of the Mediation Hypothesis

**Figure 1:**
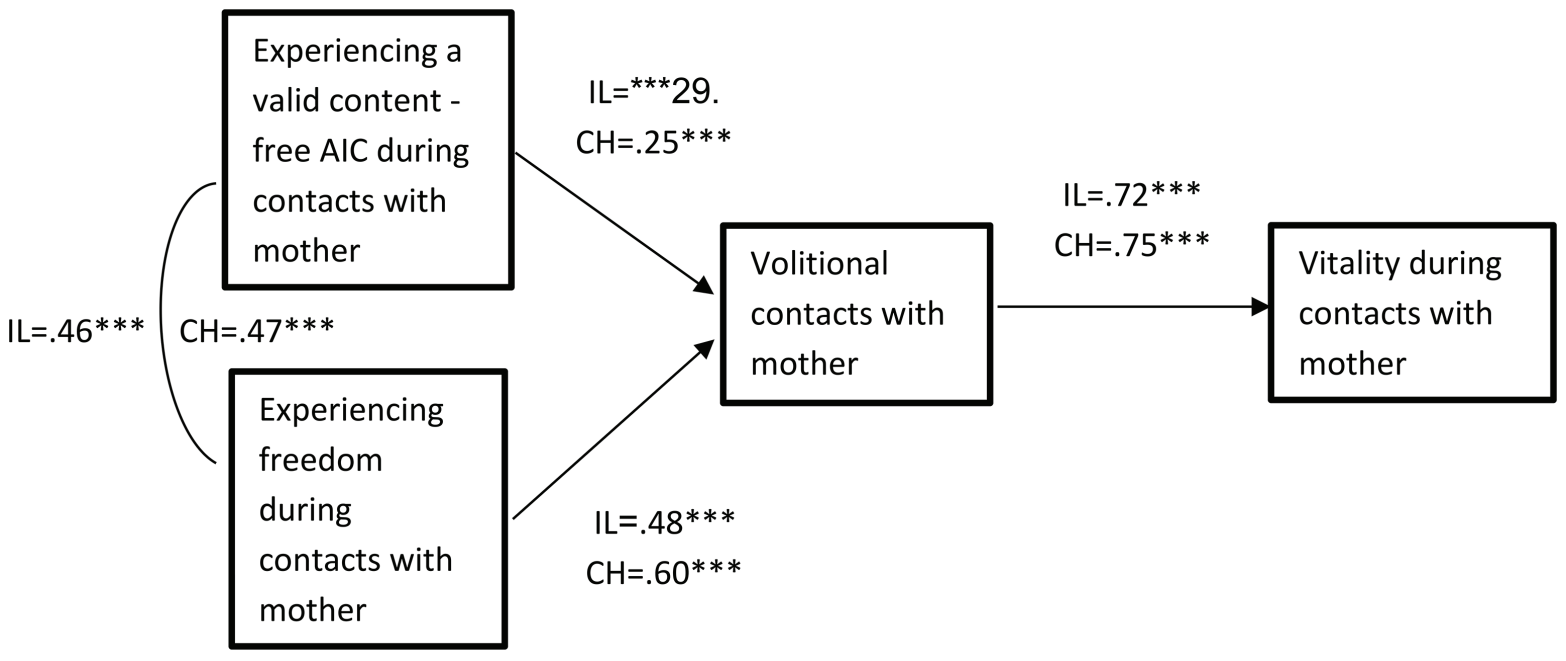
Experiencing valid content-free AIC and freedom during contacts with mother as predictors of volitional contacts and subsequent vitality in the Israeli and Chinese samples. ^*^p < 0.05; ^**^p < 0.01; ^***^p < 0.001.

To assess the mediation hypothesis, we conducted path analyses using AMOS24 (Arbuckle, 2006). The model included experiencing a valid content-free AIC and experiencing freedom as exogenous independent variables, overall volitional contacts with mother as a mediating variable; and vitality during contacts with mother as an outcome variable. Figure 1 presents the findings. The Israeli sample paths appear with the initial letter IL, while the Chinese sample paths appear with the initial letter CH. The model fit to the data was acceptable (*χ*^2^(4) = 10.80, *p* = 0.029; CFI = 0.98; RMSEA = 0.08). Thus, as expected and for both samples, experiencing a valid, content-free AIC during contacts with mother and freedom during these contacts, were positively associated with overall volitional contacts with mother, which in turn was positively associated with vitality when spending time with mother.

Bootstrapping tests showed that the mediated effects of experiencing a valid content-free AIC on vitality through volitional contacts with mother, were significant in both the Chinese sample (CI = 0.02–0.27) and the Israeli sample (CI = 0.07–0.36). Bootstrapping tests showed significant mediation effects also for experiencing freedom (Chinese sample CI = 0.18–0.53; Israeli sample CI = 0.06–0.50). The direct associations of experiencing a valid AIC and freedom with vitality, were not significant. A multi group analysis indicated that the hypothesized model was equivalent for the Israeli and the Chinese samples, and that the paths were not significantly different between these two groups (∆*χ*^2^(3) = 3.63, *p* = n.s.).

### The Moderating Role of Intrinsically Oriented AIC Content

**Figure 2:**
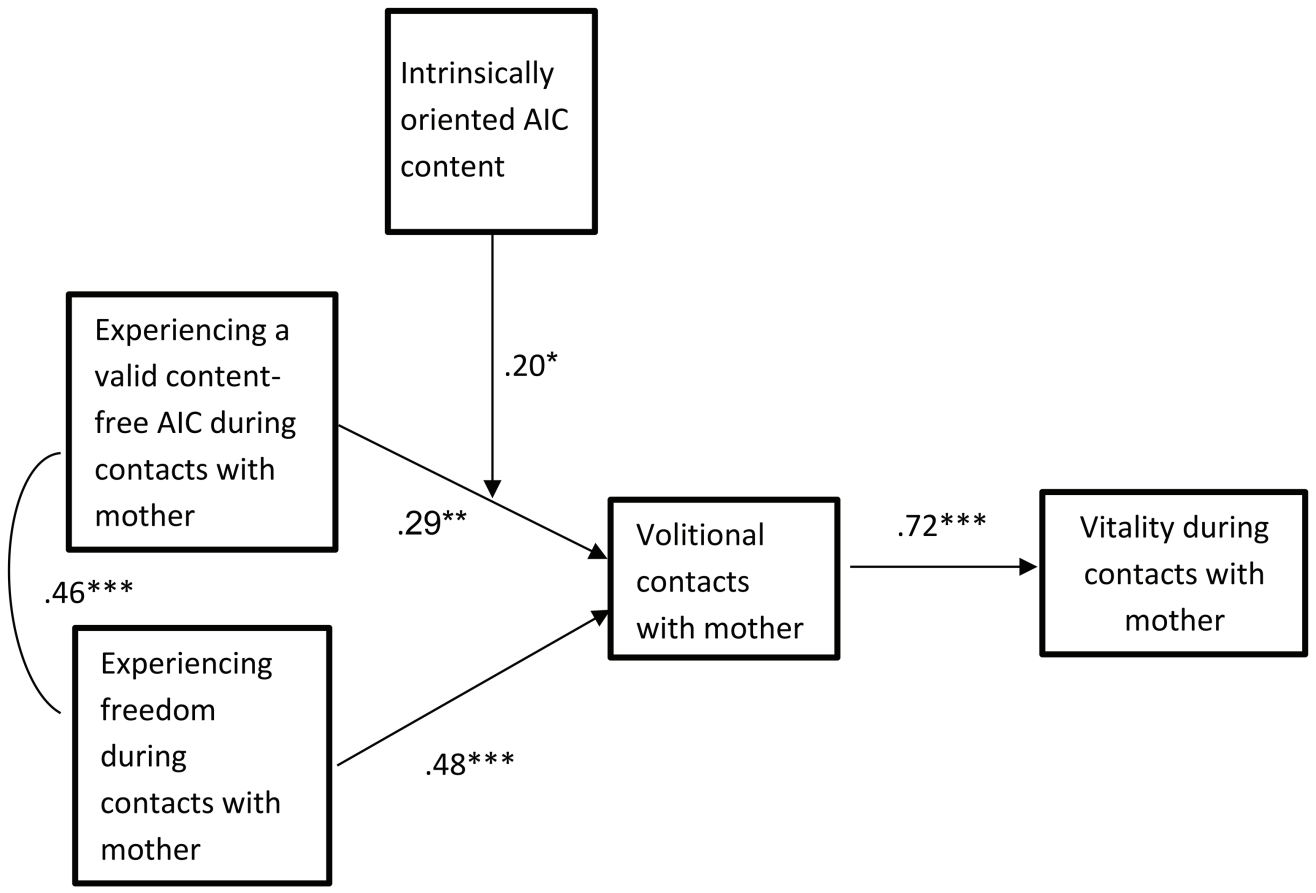
Intrinsically oriented AIC content as a moderator of the effect of experiencing content-free AIC during contacts with mother on volitional contacts and subsequent vitality in the Israeli sample. ^*^p < 0.05; ^**^p < 0.01; ^***^p < 0.001.

Structural equation modeling, using AMOS24 (Arbuckle, 2006) was conducted in order to examine whether intrinsically oriented AIC content (sense of AIC based on intrinsic aspirations) moderated the relations between experiencing a valid content-free AIC content and overall volitional contacts with mothers, and subsequent vitality during these contacts. In this analysis, experiencing a valid content-free AIC, volitional contacts, and the interaction between experiencing content-free AIC and intrinsically oriented AIC content were the independent variables. In accordance with Aiken et al. (1991), AIC, overall volitional contacts, and intrinsically oriented AIC content scores were centered. The results are presented in Figure 2. The model fit to the data was acceptable (*χ*^2^(6) = 10.12, *p* = 0.12; CFI = 0.98; RMSEA = 0.06).

**Figure 3:**
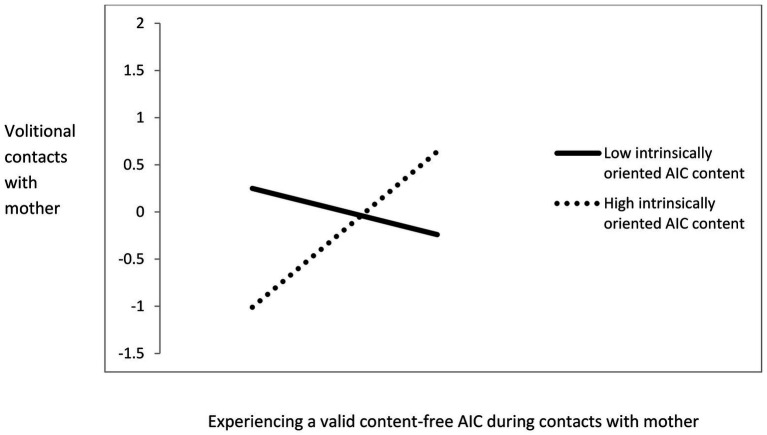
Intrinsically oriented AIC content as a moderator of the effects of experiencing a valid content-free AIC during contacts with mother on volitional contacts. Only the higher intrinsic slope is significant, *p* < 0.01.

The model indicated a significant effect of the interaction between experiencing a valid content-free AIC and intrinsically oriented AIC content on volitional contacts with mother (*β* = 0.29, *SE* = 0.32, *t* = 3.74, *p* < 0.001). Tests of simple slopes indicated that experiencing a valid content-free AIC during contacts with mother was not significantly associated with volitional contacts with mother when the level of intrinsically oriented AIC content was low (*β* = −0.06, *SE* = 0.15, *t* = −0.41, *p* = 0.68). However, when the level of intrinsically oriented AIC content was high, experiencing a valid content-free AIC during contacts with mothers was positively, moderately and significantly associated with volitional contacts with mother (*β* = 0.34, *SE* = 0.13, *t* = 2.48, *p* = 0.01). This interaction effect is presented in Figure 3. The effect of the interaction between Intrinsically oriented AIC content and overall volitional contacts with mothers on vitality was not significant (*β* = −0.01, *SE* = 0.19, *t* = −0.14, n.s.).

## Discussion

The findings of the present study indicate that in their contacts and interactions with their mothers, emerging adults distinguish between two experiences (a) having a valid, content-free, AIC, and (b) feeling free. Importantly, this distinction emerged in two widely different cultures: Israel and China. The findings are also consistent with the view that in both cultures, experiencing freedom and a valid, content-free, AIC during contacts with mothers are associated with two features indicating general need autonomy satisfaction: volitional actions, and vitality. Finally, as expected, in Jewish Israeli emerging adults, experiencing valid, content-free, AIC during contacts with mother was associated with volitional contacts with her and subsequent vitality, only when the AIC that felt validated was intrinsically oriented (i.e., based on intrinsic aspirations and goals).

The finding that in the Israeli sample, experiencing content-free AIC during contacts with mother was not associated with volitional contacts with mother and vitality - when the AIC was not intrinsic - is of special interest. First, it is interesting to try to understand possible processes accounting for the lack of salutary effects of an AIC that is not intrinsic. One possible interpretation is that perhaps, at some level, emerging adults do not really feel content and at peace with their non-intrinsic AIC, and therefore actually expect their mothers to withhold immediate confirmation of their non-intrinsic AIC, and instead raise some questions regarding the real benefits or value of such an inner compass. Assor (2018) termed this process AIC clarification.

Second, the finding that sense of AIC has beneficial effects only when the AIC is based on clearly intrinsic self-guides, may also have important implications for the assessment of AIC in future research. Thus, to the extent that this pattern will be replicated, assessment of AIC should go beyond asking people whether they have values, goals and aspirations they experience as personally important, meaningful, and potentially self-fulfilling. In addition, it is also important to determine if these self-guides are more intrinsic than extrinsic.

Results concerning mean difference between the Chinese and Israeli samples should be interpreted with caution, given that tests of scalar invariance, although generally supportive, were not fully consistent and strong. Yet, there were some clear and consistent differences that are worth noting. Thus, comparison of the means of the study variables in the two samples, indicated that the Israeli participants manifested higher means on the variables reflecting volition and experiences of freedom and validation of one’s AIC, and a lower level on the one variable reflecting lack of volition (nonvoluntary contacts with mother). This pattern is not surprising, and may reflect greater support for autonomy and authenticity in the Israeli sample compared to the Chinese one.

Notably, despite the strong differences in mean levels, the pattern of the relations observed in the two cultures was very similar. This finding is important because it suggests that *the strong difference **between** cultures, did not obscure the similar autonomy related processes posited to occur **within** each of these widely different cultures*. The findings pertaining to Chinese participants are of special interest also because they suggest that experiences of freedom and minimal control, and of having a valid AIC, are significant also in a culture that does not prioritize the values of personal autonomy and authenticity. To the extent that the findings will be replicated in future research, they seem to have important practical implications for Chinese parents. Thus, it appears that Chinese parents interested to maintain close and pleasant contacts with their emerging-adult children may do well to consider and respect their children’s need for freedom and for AIC validation.

### Questions and Implications Related to the Findings

#### Feeling Free and Having an AIC as Two Aspects of Autonomy Need Satisfaction

The findings of the present study are consistent with Assor’s (2018) view that the experience of autonomy need satisfaction in adolescence and beyond involves: (a) feeling free, and (b) feeling that one has a truly authentic inner compass (i.e., intrinsically oriented values, aspirations and goals, which are experienced as important and self-fulfilling). The idea that feeling free and having a truly authentic inner compass are aspects of a full experience of autonomy need satisfaction as viewed by Assor (2018) is supported by two findings. First, both autonomy need satisfaction aspects were associated with important expected attributes of basic need satisfaction; namely, volitional action (volitional contact and interaction with mother), and vitality. Second, these attributes emerged in widely different cultures, a pattern that is consistent with the requirement that need satisfying experiences will have similar growth promoting correlates universally.

While these findings suggest that freedom and having an AIC are important aspects of the need for autonomy, they clearly do not provide direct and rigorous support for this notion. Such support can only be obtained by experimental studies where the the satisfaction level of the two proposed aspects of the need for autonomy is manipulated, or in longitudinal research showing that satisfaction of both need autonomy aspects contribute to increased volitional action and well-being over time.

#### AIC as a Self-Organizing Vehicle, and the Need to Have Such Vehicle (or Structure)

In Ryan and Deci’s (2017) book on self-determination theory, the authors note the importance of the need for autonomy as “*a vehicle through which the **organization** of the personality proceeds, and through which other psychological needs are actualized*,” in ways that feel volitional and fully self-endorsed (p. 97). Consistent with this view, results of the present study, together with previous research (Ryan and Deci, 2017; Assor et al., 2020b), suggest that intrinsic values, aspirations and goals are structures that help organize personality and focus people on activities that feel volitional and vitalizing. Given the important role of these self-organizing decision- and action- guides, it seems reasonable to assume that as part of their general need for autonomy, and beginning in adolescence, people need to feel that they have such self-organizing structures; namely, a need to feel that they have an authentic inner compass comprised of deeply integrated, authentic intrinsic values, aspirations and goals.

From this perspective, the conception of the experience of having a valid AIC as part of the general need for autonomy allows us to understand the role of deeply integrated intrinsic values and aspirations in optimal functioning in a somewhat different light. Specifically, consistent with Assor (2018), it appears that integrated *intrinsic values, aspirations and goals are not only vehicles for optimal need satisfaction*. Rather, starting in youth and beyond, the experience of having such deeply integrated values and aspirations may be an important component of the experience of autonomy need satisfaction. Put differently, as *part of their need for autonomy and beginning in adolescence, people need to feel that they have intrinsic, autonomously formed, self-directing and decision-guiding, values, aspirations and goals*. Accordingly, beginning in adolescence, when people do not feel that they have AIC (integrated intrinsic values, aspirations and goals), they are less likely to feel that their need for autonomy is deeply and optimally satisfied.

#### Comparing the Relations of Feeling Free and Experiencing a Valid AIC With Volitional Contacts and Vitality

As shown in Table 2, in both samples, the experience of feeling free during interactions with mother had higher negative correlations with nonvoluntary contacts with mother than the experience of having a valid AIC. These differences were significant in both samples. Interestingly, feeling free and AIC did not have consistently different correlations with other variables. The finding that feeling free had particularly strong negative associations with nonvoluntary contacts (including controlled, a-motivated and resistant contacts) is consistent with Assor’s (2012, 2018) assumption that the experience of freedom from control and coercion is a fundamental component of the need for autonomy, and therefore the thwarting of this striving leads to particularly problematic relational and affective outcomes.

#### The Relations Between Experiences of Freedom and Volition as Important Indicators of Autonomy Need Satisfaction

In addition to AIC, the present study also focused on the experience of freedom as an essential component of autonomy need satisfaction. SDT conceptualizations and measures of autonomy usually include terms similar to the concept of freedom. For example, psychological freedom (Basic Psychological Need Satisfaction and Frustration Scale [BPNSFS], 2018), avoidance of heteronomous control or coercion (Deci and Ryan, 2000; Ryan and Deci, 2017) and similar terms (Ryan and Deci, 2006; Weinstein and Ryan, 2010). However, these conceptualizations and measures also use the concept of volition to describe experiences of need autonomy satisfaction (e.g., Deci and Ryan, 2000; Ryan and Deci, 2017). Therefore, it seems reasonable to examine whether the terms volition and freedom refer to the same experience. If this is the case, of course, the concept of freedom is unnecessary, and we should simply substitute it with the concept of volition.

In here, we propose that both concepts are useful, because they describe phenomena that although related, are analytically and psychologically distinct. Sense of true volition describes the ***overall experience*** of autonomy need satisfaction, regarding an action (or a context or a period in ones’ life, as well as regarding having specific thoughts, feelings, goals, and plans). This sense of volition is often conceptualized in SDT by the notion of relative autonomy, and is assessed by an index in which controlled motivations, and sometimes also a-motivation and defiance/resistance are subtracted from autonomous motivations. In the present study, sense of volition was assessed *via* the measure of volitional contacts. Unlike the general concept of volition, the reality and experience of freedom refers to ***specific experiences*** that people need in order to feel a sense of volition. Thus, when people feel coerced to do certain things, this is likely to harm their sense of volition regarding the relevant action or context. In contrast, when people feel that they are free to experience and, explore things that they may find valuable, this is likely to increase their sense of volition, because they feel that there is a chance that actions freely engaged will be satisfying.

However, being free from coercion and free to act is only one factor that contributes to sense of volition. Thus, people may be free to act, but still not feel a sense of volition regarding any action because they do not know what is important to them, or they do not believe they can find what is important to them. Therefore, a second **specific** need autonomy component that has to be satisfied in order to feel a sense of volition is knowing what is truly important to you (i.e., having an AIC). Then, sense of volition regarding a certain action or context is further increased when people express in their behavior what is truly important to them. The results of the present study are consistent with the above view of volition, freedom and AIC, because they suggest that experiencing both freedom and a valid AIC may enhance the sense of volitional action, a widely accepted indicator of autonomy need satisfaction. Further studies will have to examine the extent to which the relations observed are indeed causal.

### Methodological Limitations and Directions for Future Research

The present study has a number of limitations, which may be addressed in future research. First, because of the cross-sectional nature of the design, it was not possible to draw causal inferences. Future research should employ longitudinal or experimental designs which may allow causal inferences. Second, it is important to examine if the results will hold in larger samples. Third, given that support for scalar invariance of measures across the two samples was only moderate, future research may attempt to develop items that will show a higher level of equivalence across cultures. Fourth, the assessment of the degree to which the goals and aspirations underlying sense of AIC are intrinsic, should be refined and improved. The measure used had the important advantage of relying on responses generated by the participants. However, the range of values obtained was narrow, and it may be useful to supplement this measure with standard scales of intrinsic vs. extrinsic goals. After participants complete these scales, it might be useful to ask them to select the aspirations that most reflect who they are and want to be, guide important decisions, and determine their direction and plans in life.

Fifth, it is important to examine whether the results will be replicated for experiences of freedom and having a valid AIC with fathers, and other significant others such as friends and romantic partners. Sixth, it important to examine if children’s experiences of their mother correspond with their mother’s self-perception on these issues. Seventh, future research may examine whether the autonomy experiences of emerging adults during their contacts with their mother actually affect mother experiences and reactions during such contacts. Eighth, it is interesting to examine whether, across time, lower levels of volitional contacts with mother result in actual decreased contact, perhaps even detachment. Ninth, it is important to examine if the pattern observed in the present study is replicated in other cultures than the two examined here. Finally, it is important to examine if the experiences of freedom and having a valid AIC have similar effects also in relationships and contexts differing from those examined in the present study, for example, teacher-student relationship, or manger – employee relationship.

## Conclusion

The results of the present study are consistent with the view that there are two kinds of specific experiences that contribute to the satisfaction of the general need for autonomy in emerging adults, as reflected in volitional, self-endorsed, and vitalizing actions. These experiences are: (1) feeling free, and (2) feeling that one has a valid authentic inner compass (AIC). The results also suggest that experiencing one’s AIC as valid during contacts with mother increases the motivation to spend time with her and subsequent vitality, only if one’s sense of having an AIC is based on aspirations and goals that are intrinsically oriented.

## Data Availability Statement

The raw data supporting the conclusions of this article will be made available by the authors, without undue reservation.

## Ethics Statement

The studies involving human participants were reviewed and approved by Human Subjects Research Committee - Ben Gurion University Request Sub-Number: 5104-1. The patients/participants provided their written informed consent to participate in this study.

## Author Contributions

All authors listed have made a substantial, direct, and intellectual contribution to the work and approved it for publication.

## Funding

This study was funded by Israel Science Foundation research grant to the first author.

## Conflict of Interest

The authors declare that the research was conducted in the absence of any commercial or financial relationships that could be construed as a potential conflict of interest.

## Publisher’s Note

All claims expressed in this article are solely those of the authors and do not necessarily represent those of their affiliated organizations, or those of the publisher, the editors and the reviewers. Any product that may be evaluated in this article, or claim that may be made by its manufacturer, is not guaranteed or endorsed by the publisher.
